# [Expression of Concern] ETS-1: A potential target of glycolysis for metabolic therapy by regulating glucose metabolism in pancreatic cancer

**DOI:** 10.3892/ijo.2025.5786

**Published:** 2025-08-11

**Authors:** Xiu Zhang, Dan Wu, Mohanad Aldarouish, Xiaodong Yin, Chunyan Li, Cailian Wang

Int J Oncol 50: 232-240, 2017; DOI: 10.3892/ijo.2016.3770

Following the publication of the above paper, it was drawn to the Editor's attention by a concerned reader that, regarding the western blots shown in Figs. 4B and 5B, the AMPK panel in Fig. 4B looked strikingly similar to the ATG5 panel in Fig. 5B, and the p-AMPK panel in Fig. 4B looked highly similar to the ATG7 panel in Fig. 5B.

The authors were contacted by the Editorial Office to offer an explanation for these apparent anomalies in the presentation of the data in this paper, although up to this time, no response from them has been forthcoming. Owing to the fact that the Editorial Office has been made aware of potential issues surrounding the scientific integrity of this paper, we are issuing an Expression of Concern to notify readers of this potential problem while the Editorial Office conitnues to investigate this matter further.

